# Glial cells are involved in day–night protein dysregulation in the hippocampus of a mouse model of Alzheimer’s disease

**DOI:** 10.1038/s41598-025-30965-8

**Published:** 2025-12-04

**Authors:** Aurélien M. Badina, Hamza Tahiri, Ali Ouarour, Kelly Ceyzériat, Philippe Millet, Benjamin B. Tournier, Ibtissam Chakir

**Affiliations:** 1 Department of Psychiatry, Faculty of Medicine, University of Geneva, Geneva, Switzerland; 2 Faculty of Science, Laboratory of Biology and Health, Abdelmalek Essaâdi University, Av. Khenifra, Tetouan, 93000 Morocco; 3 Department of Biology and Geology, Sultan Moulay Slimane University, Beni-Mellal, Morocco; 4 CIBM Center for BioMedical Imaging, Geneva, Switzerland; 5 Department of Psychiatry, University Hospitals of Geneva, Avenue de la Roseraie, 64, Geneva, 1206 Switzerland; 6 Département de Biologie et Géologie FP Béni Mellal, USMS, Béni Mellal, Morocco

**Keywords:** 3xTgAD, Astrocytes, Electron transport chain, Complex I, Metabolism, Cell biology, Neuroscience

## Abstract

The hippocampus regulates memory and cognition, both of which are influenced by circadian rhythms. These rhythms also drive time-dependent gene expression in neurons and glial cells, affecting hippocampal function. In Alzheimer’s disease (AD), alterations in these rhythms, contributing to cognitive decline, is little understood. This study examined hippocampal proteomes from 7-month-old wild-type (WT) and 3xTgAD mice at two time points (Zeitgeber 2, ZT2 and ZT14) to detect early pathological changes. In WT mice, 199 proteins (8%) showed diurnal variation, particularly those linked to energy metabolism and neuronal/glial functions. In 3xTgAD mice, this rhythmic variation dropped by half (3.6%), with only five proteins shared across genotypes. Moreover, significant differences in protein expression emerged between WT and 3xTgAD at both time points, notably involving mitochondrial function, oxidative phosphorylation, and ATP production. Functional clustering revealed disrupted bioenergetic pathways, especially affecting complex I of the electron transport chain and astrocytic metabolism. These early alterations suggest a breakdown in daily control of hippocampal energy regulation in AD. The results highlight the critical role of sampling time in research and suggest that circadian disruption in astrocytic and neuronal metabolism may play a central role in AD progression and could inform future chronotherapeutic approaches.

**Supplementary Information:**

The online version contains supplementary material available at 10.1038/s41598-025-30965-8.

## Introduction

The hippocampus is one of the key brain regions controlling cognitive functions and memory systems. These cognitive functions include learning capacities, synaptic plasticity, neuron firing, long-term potentiation, and all exhibit diurnal variations^1–3^. The control of physiological functions is ensured by the existence of a central clock synchronized at 24 h by the light/dark cycle. This circadian clock located in the suprachiasmatic nuclei (SCN) is an internal biological mechanism that regulates hormonal cycles, gene expression and cellular function over a period of around 24 h. These endogenous and self-maintained rhythms are generated by circadian variation in expression and feedback loops of “clock genes”. The master circadian clock drives the phase of the other secondary clocks dispersed in the body, including various brain areas and peripheral organs, via endocrine and neuronal pathways. Target tissues express clock genes, enabling local function control over 24 h through synchronization with the SCN. In the hippocampus, gene expression also shows 24-hour variations and are thought to be at the root of the daily fluctuations in hippocampus-dependent cognitive abilities^4–7^. In addition to clock genes, in the ventral hippocampus of mice, 170 proteins out of 1500 analysed (11%) show daily variations^2^. In the rat hippocampus, RNA sequencing showed the expression of 485 genes to be rhythm-dependent^8^. In addition to neurons, glial cell activity is also dependent on the time of day. Microglia and astrocytes have 24-hour rhythms regulating their morphology, the expression of their transcriptome and proteome and their inflammatory response^9–11^. In the hippocampus, microglia, astrocytes and their alterations linked to circadian rhythms modulate memory capacity^12,13^.

The hippocampus is also one of the main areas involved in Alzheimer’s disease (AD). Both humans and rodent models of AD, such as the 3xTgAD mouse model, present cognitive dysfunctions, neuroinflammation, and the accumulation of both extracellular amyloid-β (Aβ) deposits and intracellular phosphorylated forms of Tau. In the hippocampus of the 3xTgAD mouse model, Aβ and Tau start aggregating at around 6-month-old, and plaques are visible at 12-month-old^14–16^. Protein aggregation eventually alters glial cell function, starting in early stages of the disease^15,16^. Indeed, homeostatic glial cells endorse a neuronal support role but also protection. Astrocytes supply neurons with lactate for energy, are involved in synaptic transmission and generally contribute to injury or disease response. Microglia are the brain immune residents, involved in synaptic plasticity as well as tissue repair and immune surveillance. In AD, a proteomic study in the mouse cortex showed an activation of astrocytes and microglia leading to an altered immune response and lipid metabolism^17^. Glial cell reactivity in the hippocampus is also known to be involved in cognitive impairment^18^. Reactive astrocytes alter their own metabolism, and it has been shown to impact neurons and cognitive performances, while microglial activation has been shown to be toxic to local brain environment and neurons^19,20^. In addition, the 24 h-rhythm of cognitive performance, neuronal excitability and clock gene expression in the hippocampus are altered in AD^21,22^. As an example, wild-type (WT) mice show higher cognitive performances during the dark phase, but these phases are desynchronized in AD models^21,23^. At the level of the central clock, neuronal activity shows a lower amplitude of day/night variations in an AD model^24^, suggesting an alteration of daily pathways. In agreement with this idea, disturbances in the rhythms of activity to rest cycles, temperature and hormonal regulations are observed in AD, including in the 3xTgAD mouse model^25,26^. At the pathway and molecular level, little is known about rhythm alterations in the hippocampus.

Considering the impact of circadian rhythms on neurons and glial cells, and the consequences of dysregulated glial cells on metabolism, inflammation and eventually neurons, we investigated the crosslink between the two. In this study, we used proteomics to measure protein density in the hippocampus of WT and 3xTgAD mice at two different zeitgeber time (ZT). ZT0 is defined as the time when lights went on. We analysed the proteome at ZT2 and ZT14, symbolizing the onset of resting and active phases of the animals. The quantification of proteins was preferred as it has been shown that mRNA quantities do not always fluctuate in phase with proteins^27^. To highlight early alterations in the pathology, this analysis was carried out on hippocampi from 7-month-old mice. At this age, the mice show slight cognitive and neurochemical alterations, representing the early stages of human pathology.

## Results

### Proteomic profile at ZT2 versus ZT14

**Fig. 1:**
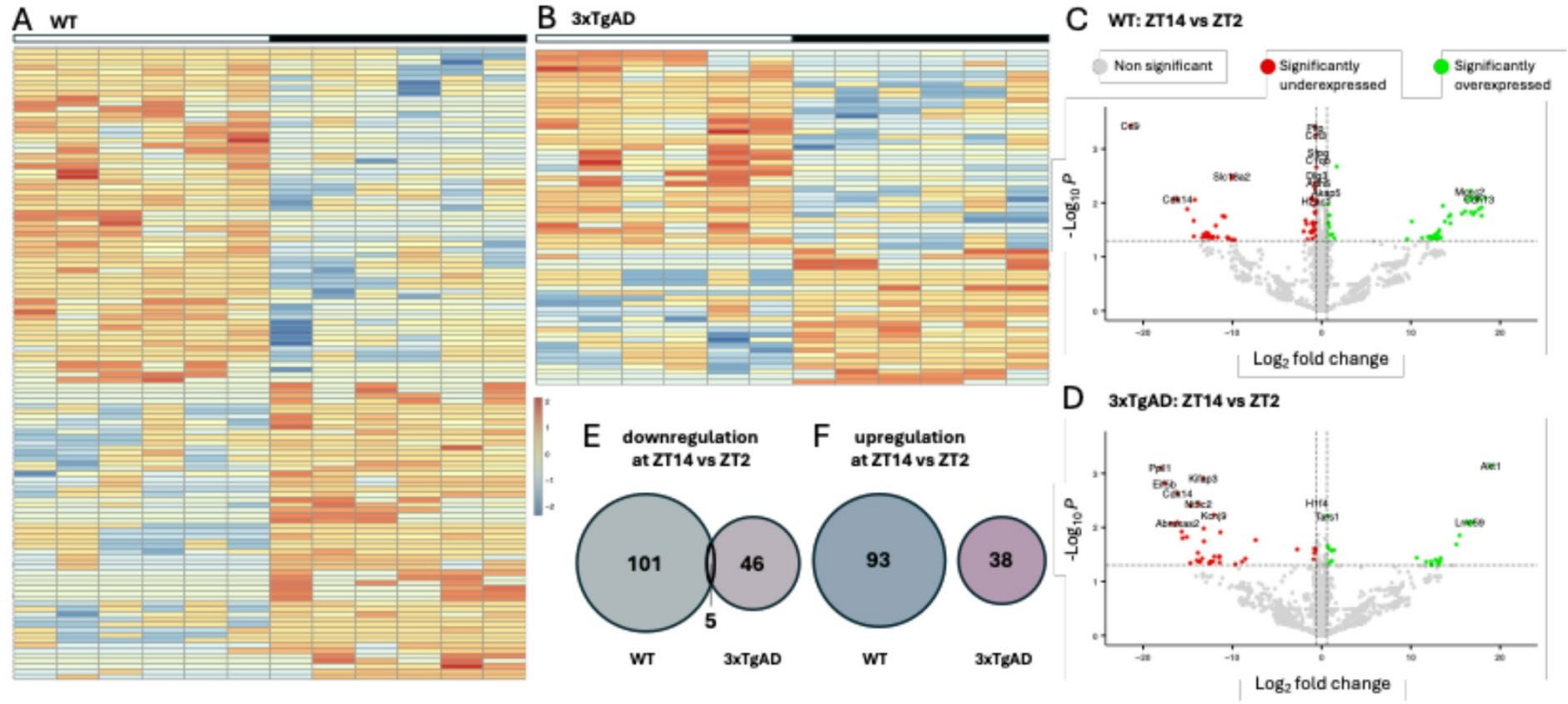
Zeitgeber time-dependent protein density in WT and 3xTgAD mice. (**A**, **B**) Heat-map of protein density at ZT2 (white bar) and ZT14 (black bar) in WT (**A**) and 3xTgAD (**B**). Each column represents data for one animal, and each row represents one protein. Only proteins that are significantly different between ZT2 and ZT14 are shown. The color scale represents row-wise z-scores of normalized log2 intensities. 0 equals the protein abundance mean across the plotted samples, and positive or negative values indicate standard deviations above or below that mean. (**C**, **D**) Volcano plot of the differential density expression in the hippocampus of WT (**C**) and 3xTgAD (**D**), at ZT2 as compared to ZT14. Dots represent individual protein whose expression is downregulated (red) or upregulated at ZT14 (green) and unaltered (grey, |log2FC| ≤ 0.58 or *P* value > 0.05) as compared to ZT2. (**E**, **F**) Genotype-specific differential protein expression, and overlap, downregulated at ZT14 vs. ZT12 (**E**) and upregulated at ZT14 vs. ZT12 (**F**) in.WT (blue) and 3xTgAD (pink) mice.

Differential protein expression between ZT2 and ZT14 of the WT proteome shows that out of 2460 proteins analysed, 199 are differentially expressed proteins (DEP, 8% of total proteins) with 106 downregulated and 93 overexpressed at ZT14 vs. ZT2 (Fig. 1A–D and Supp data 1). These DEP are involved in mitochondrial energy metabolism (ZT2 > ZT14: COX1, ND5, NDUFB5, NDUFB10, NDUFS2, SDHAF2, ATP5MJ, UQCRC1, MPC2, SLC25A13, SLC25A18, AK2, AK3 and VDAC2), glucose metabolism (ZT2 > ZT14: PCX, ZT2 < ZT14: ALDOA, PGM1), nucleotide metabolism (ZT2 > ZT14: CTPS1 and SAMHD1), lipid and steroid metabolism (ZT2 > ZT14: PLPP3, DPM1 and HSD17B11), Krebs cycle (ZT2 > ZT14: SHMT2, ZT2 < ZT14: IDH1 and MCCC2), and oxidative stress and cellular detoxification (ZT2 > ZT14: ADH5 and SIRT2, ZT2 < ZT14: GCLC, GLRX5 and OPLAH).

The identified DEPs indicate involvement of multiple specific cell types, highlighted by their preferential gene expression patterns. Representative changes including GABRG2 and RYR2 in neurons, SLC1A3 and NDRG2 in astrocytes, C1QB in microglia, and MOG and PLP1 in oligodendrocytes, among others, illustrate that circadian regulation extends across neuronal and glial populations (See Supp data 1 for the full list of proteins).

In 3xTgAD mice, the variation in protein density between ZT2 and ZT14 is reduced by half. Indeed, only 89 proteins showed a differential expression (3.6% of proteins) with 51 downregulated proteins and 38 overexpressed at ZT14 vs. ZT2 (Fig. 1B,D and Supp data 1). The heat-maps underline that a greater number of proteins show ZT-dependent expression in WT mice than in AD mice, highlighting daily rhythm dysregulation in AD. These proteins are involved in fatty acid metabolism (ZT2 < 14: ALDH1A1 and FABP3), nucleotide metabolism (ZT2 > 14: NT5C2 and PRPS1L3), protein synthesis (ZT2 > 14: CARS1, ZT2 < 14: TARS1, LARS1), cellular death/survival (ZT2 > 14: ATG5, ZT2 < 14: AKT1), and oxidative stress (ZT2 > 14: PRDX1).

A comparison of the protein lists showing ZT2 vs. ZT14 differences between WT and 3xTgAD mice reveals that only 5 proteins are shared, all showing higher levels at ZT2 (Fig. 1E,F). These proteins are mainly associated with oligodendrocytes and neurons (MAG, CNP, CDK14, SNRNP200, RAP1GDS1).

### Dysregulation of the proteomic profile in 3xTgAD

**Fig. 2:**
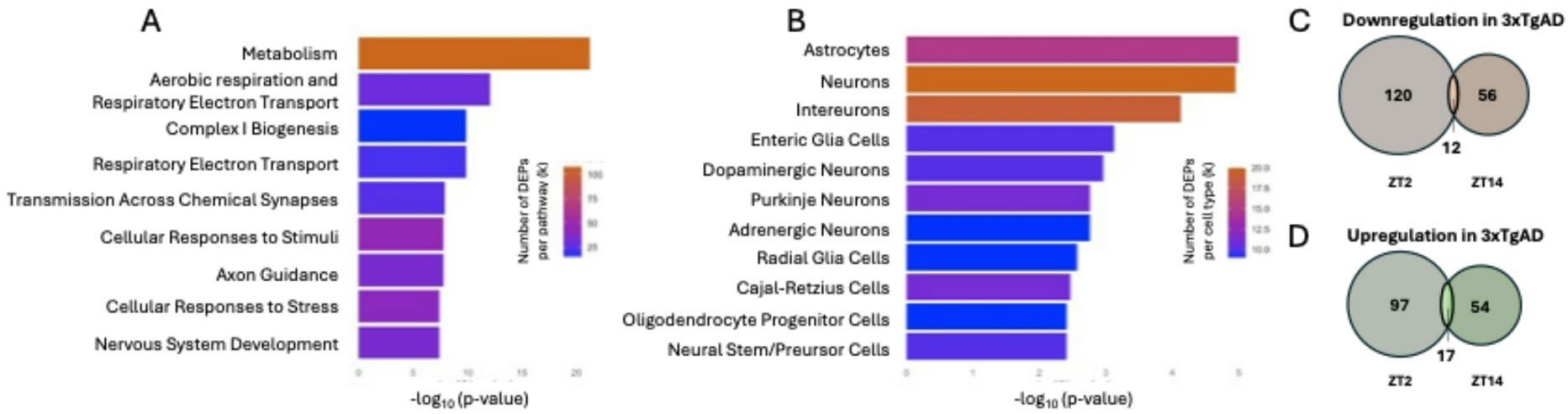
Pathways and cell types involved in alterations of the hippocampus in 3xTgAD. (**A**, **B**) Over-representation analysis (ORA) of differentially expressed proteins (DEPs) in 3xTgAD vs. WT. (**A**) Number of dysregulated proteins involved in the displayed pathways, queried against the Reactome Pathway Database. (**B**) Number of dysregulated proteins specific to the displayed cell types, using PanglaoDB markers.Bars show − log10 p-value (FDR; hypergeometric test with Benjamini–Hochberg correction), using all quantified proteins as background (*n* = 2,460); the color scale indicates the number of DEPs annotated to each term (k). (**C**) In 3xTgAD mice, 132 and 68 proteins were downregulated at ZT2 and ZT14 compared to WT, respectively. (**D**) In 3xTgAD mice, 114 and 77 proteins were upregulated at ZT2 and ZT14 compared to WT, respectively.

At ZT2, there were 246 DEP in 3xTgAD compared to WT, with 132 underexpressed proteins and 114 overexpressed proteins. At ZT14, the differences were slightly less pronounced, with a total of 139 DEP containing 68 underexpressed proteins and 71 overexpressed proteins in 3xTgAD mice. Among the main impacted pathways, there is the energetic metabolism (108 proteins), mitochondrial processes (30 proteins) and cell responses to stress and stimuli (45 proteins, Fig. 2A and Supp. Data 2). Astrocytes, neurons and interneurons are the most impacted cells (between 15 and 20 proteins involved, Fig. 2B and Supp. Data 3). The same analysis focused on proteins modified at ZT2 revealed the same functional categories and cell-type, with neurons being more represented than astrocytes (Supp. Data 7–8). At ZT14, mitochondrial involvement was reduced (9 proteins), and unlike astrocytes, neither neurons nor interneurons reached significance (adj. p = < 0.001, 0.25 and 0.069 respectively, Supp. Data 9–10).Among the underexpressed proteins, only 12 were identical between ZT2 and ZT14, while only 17 proteins were overexpressed at ZT2 and ZT14 in 3xTgAD mice compared to WT (Fig. 2C,D), demonstrating the elevated importance of ZT in the 3xTgAD vs. WT comparison.

We then performed functional annotation clustering on all DEP between 3xTgAD and WT (Table 1). DAVID annotation cluster 1 (Enrichment Score = 12.5) revealed a highly significant enrichment of mitochondrial components and targeting signals. Notably, 67 genes were associated with the keyword ‘mitochondrion’, and over 30 contained mitochondrial transit peptides, indicating active mitochondrial import. The presence of matrix-localized proteins suggests metabolic functions within the organelle, supporting the role of mitochondrial dynamics and bioenergetics. Annotation cluster 2 (Enrichment Score = 7.78) was strongly enriched in functions related to the mitochondrial electron transport chain, particularly complex I. Key annotations included ‘mitochondrial inner membrane’, ‘NADH dehydrogenase activity’, and ‘oxidative phosphorylation’. Additionally, several genes were associated with KEGG pathways relevant to neurodegenerative diseases. These results highlight a functional convergence toward mitochondrial bioenergetics and its pathological disruption in AD. Annotation cluster 3 (Enrichment Score = 4.61) highlighted a significant overrepresentation of ATP- and nucleotide-binding proteins. Forty-seven genes were annotated with the GO term ‘ATP binding’, suggesting that a large portion of the gene set is involved in energy-dependent molecular functions. This functional enrichment further supports the dysregulation of mitochondrial energetics and ATP turnover in 3xTgAD mice. Annotation cluster 4 (Enrichment Score = 3.8) grouped genes associated with the microtubule cytoskeleton and microtubule-binding activities. This included 16 genes linked to the structural GO term ‘microtubule’ and 12 with ‘microtubule binding’ activity, suggesting a potential role in intracellular transport or cytoskeletal dynamics. Details of the DAVID annotation clusters (from 1 to 68) are given in Supp. Data 4.

**Table 1:** Summary of the david’s functional annotation clustering. *ES* enrichment score.

Cluster	ES	Main functional theme	Representative enriched terms
1	12.5	Mitochondrial targeting and localization	Mitochondrion, Transit peptide, Mitochondrial matrix
2	7.78	Mitochondrial respiratory chain & neurodegeneration	Complex I, ATP synthesis, Oxidative phosphorylation, Neurodegeneration
3	4.61	ATP-dependent molecular functions	ATP binding, Nucleotide-binding
4	3.8	Microtubule cytoskeleton and intracellular transport	Microtubule, Microtubule cytoskeleton, Microtubule binding

**Fig. 3:**
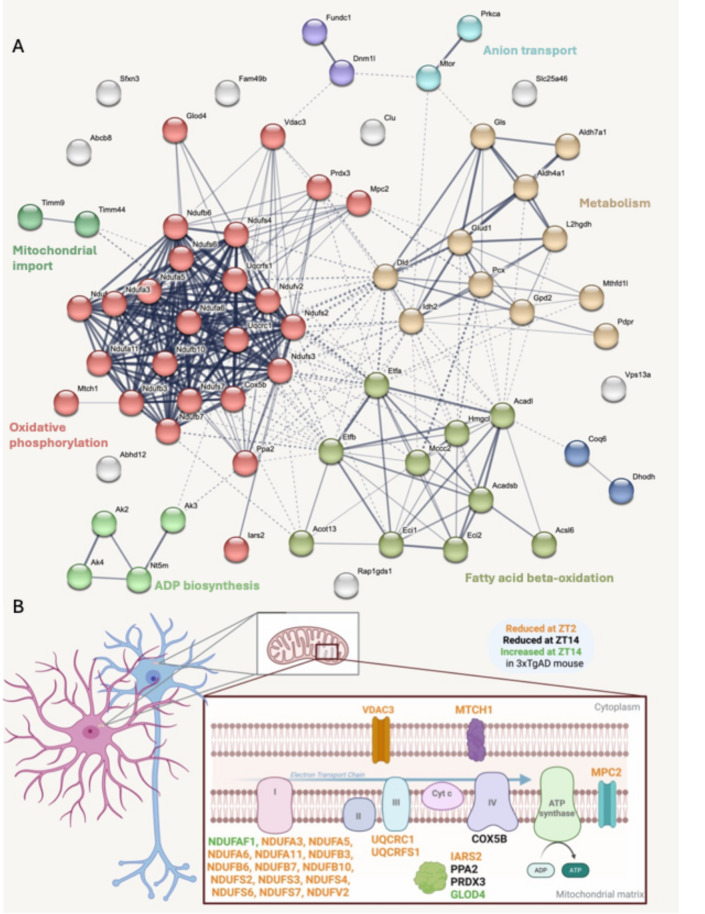
Protein-protein interaction network. (**A**) STRING-based protein interaction network (Markov Cluster Algorithm) of the 69 proteins from the DAVID annotation cluster 1. Each node represents a protein; edges represent interactions based on both functional and physical protein associations. Proteins were grouped into functional clusters (color-coded) reflecting biological modules (oxidative phosphorylation, ADP biosynthesis, etc.). Clustering inflation parameter: 3.0, Interaction score 0.4. Dotted line: edges between clusters. Edge thickness: strength of data support. (**B**) Diagram depicting the sub-mitochondrial localization of the 25 proteins from module 1 (red in A) involved in mitochondrial AD dysregulation. The Fig. 3B was made with Biorender.

From the 69 proteins of the main group (the DAVID annotation cluster 1), the MCL clustering identified 4 major functional modules involved in oxidative phosphorylation and complex I biogenesis (25 genes), metabolism (dicarboxylic acid and carbon metabolism, glycolysis and glucogenogenesis, 11 genes), fatty acid beta-oxidation (10 genes), and finally, ADP biosynthesis (4 genes) (Fig. 3A and Supp. Data 5). The main module, composed of 25 proteins, is involved in the electron transport chain for the regulation of ATP synthesis. 18 of them are directly linked to the complex I, III and IV (see Fig. 3B or Supp. Data 6 for the full names of these proteins). The others could be involved in the direct or indirect regulation of energy production. In fact, the mitochondrial Isoleucyl-tRNA synthetase 2 (IARS2) plays a role in the translation of electron transport chain complex proteins. The mitochondrial pyruvate carrier 2 (MPC2) is involved in the mitochondrial entry of pyruvate, whose transformation by the Krebs cycle produces the main source of cellular ATP and also NADH, the entry path of the electron transport chain. The Peroxiredoxin 3 (PRDX3) reduces hydrogen peroxide induced by the functioning of the electron transport chain. Other proteins may be involved in regulating oxidative stress (by-product of the electron transport chain), such as Glyoxalase domain-containing protein 4 (GLOD4) and Voltage-dependent anion channel 3 (VDAC3), but the links are less clear. The mitochondrial Pyrophosphatase 2 (PPA2) regulates inorganic phosphate availability and supports ATP-consuming biosynthetic reactions. Its dysfunction disrupts ATP dynamics and mitochondrial energy cycles.

## Discussion

We show that 8% of proteins are differentially expressed between ZT2 and ZT14 in WT, and that this effect is reduced to less than 4% in the 3xTgAD model. Only 5 proteins are common between the two genotypes, showing profound variations in 3xTgAD. The most striking daily effect is about the density of proteins involved in the electron transport chain (ETC): at ZT2, 3xTgAD mice showed lower levels of 10 proteins directly or indirectly involved in ATP-energy synthesis. All cell types are affected, particularly astrocytes. These data show that the timing of sampling, euthanasia or observation is fundamental in future studies of the hippocampus and early AD.

In WT mice, the main expression differences between ZT2 and ZT14 are carried by genes involved in the general regulation of metabolism (glucose, nucleotide, lipid and steroid metabolisms) and energy control (mitochondrial energy, Krebs cycle and oxidative stress). We cannot rule out that preferential food intake during the nocturnal phase contributes to this difference within this group. However, our observations in the hippocampus are in line with literature data showing daily rhythms in hippocampal functions such as memory^1–3^. In 3xTgAD mice, DEP between ZT2 and ZT14 are reduced by more than half, indicating a disruption of hippocampal rhythmic activity in these mice. In addition, there is a change in the functional expression of these rhythms: proteins involved in glucose, lipid and steroid metabolism are no longer represented. This points to a dysregulation of hippocampal function that may be linked to the onset of neuroinflammatory reaction and accumulation of soluble forms of amyloid^15,16,28^. Links between the presence of Aβ and disruption of cellular metabolism, including glycolysis and energy production, have been reported in literature^19,29,30^. In addition, our data show that the hippocampal function is more disrupted in the early resting phase than in the early active period in 3xTgAD mice, as compared to WT, and mainly involved astrocytes, metabolism and mitochondria.

The involvement of mitochondria in AD has been widely described, and their dysfunctions can lead to neuronal death^31,32^. Mitochondria are involved in ATP synthesis to supply cells with energy and in regulating the production of reactive oxygen species (ROS). ROS is a byproduct of the ETC when regenerating substrates for the Krebs cycle, and is made of 5 complexes, starting with complex 1 made up of 45 proteins and ending with the ATP synthase. A disturbance in the density of some of the proteins making up complex I was observed in the human medial frontal gyrus^33^, but this study was only carried out on 4 samples (2 CT, 2 AD) from a mixture of different subjects, which limits the conclusions. The presence of Aβ in mitochondria leads to alteration of ETC^33,34^, which may result in increased ROS, as observed in AD^35^. In fact, the overproduction of ROS is associated with the dysfunction of complexes, in particular complex I^36,37^. We extend these observations with the presence of time-dependent regulation of the difference. Indeed, we showed a significant decrease in complex I proteins at ZT2. This observation has important implications: 1/considering the time of hippocampal sampling in preclinical studies of AD and integrating the time of death in human *postmortem* studies is necessary to estimate the effects. 2/even though the hippocampus does not have a self-sustaining clock, it shows variations in day/night function. Cognitive reinforcement strategies used as treatment should therefore consider this effect, to achieve chronotherapy. Alterations in other complexes, such as complex IV, have been reported, suggesting that the entire ETC chain is altered in AD^38^. Future studies should therefore focus on the functioning of the ETC as a whole and ATP production, to better understand the alterations.

It is highly likely that dysregulation of metabolism and mitochondria induces alterations in cell function. Interestingly, the number of proteins involved in the ZT2 vs. ZT14 difference is reduced by about half for neurons (from 12 to 7) and oligodendrocytes (from 6 to 4) and by 75% for astrocytes (from 11 to 3). For microglia, it is more difficult to conclude, as only 2 and 0 specific microglial proteins are observed in WT and 3xTgAD mice, respectively. Astrocytes play a role in the regulation of hippocampal functions and are involved in the control of Aβ accumulation, neuronal activity and pathological progression^18,39–42^. Changes in gene expression profiles are observed with the progression of the pathology, including the emergence of specific astrocyte subtypes whose functions remain uncertain, as well as an increase in astrocytic reactivity that may exacerbate the disease^43–46^. Previously, by modulating astrocyte function, we and other groups have been able to reduce disease-related symptoms^18,19,28,47,48^. Importantly, astrocytes possess functional circadian clocks that may contribute to the regulation of daily neuronal activity^49^. While most studies have examined astrocyte–neuron interactions in the suprachiasmatic nucleus (SCN)^50,51^, our work supports a role for astrocytic circadian rhythms in the hippocampus. Interestingly, disruption of circadian rhythms can also influence the temporal progression of pathology. Indeed, rhythm alterations that disturb sleep are associated with increased neurodegeneration^52,53^. Although the underlying mechanisms are not yet fully understood, such disturbances are likely to enhance ROS production, promote neuroinflammation, and impair glymphatic activity^52,53^. Notably, astrocyte function is involved in all three of these processes, and it has been proposed that the daily regulation of astrocytes serves as a protective mechanism for neurons^50^. Astrocytes therefore represent a promising therapeutic target, and our findings support this concept. Our observation of the particular involvement of astrocytes has several implications. First, it confirms their early role in metabolic dysfunction^29^ and AD in general^54–56^. Second, it is important to point out that the role of mitochondria in astrocytes is different than in other cell types. Indeed, astrocytes with inhibited mitochondria do not show disorders, suggesting that the energy linked to mitochondrial ATP production is not indispensable to astrocytes^57,58^. Moreover, inhibition of pyruvate entry into astrocyte mitochondria - one of the initial pathways of ETC - improves the neurochemical symptoms of the pathology^19^. Thus, although mitochondrial activity is not necessary for astrocyte function, and blocking some of its activity may even be beneficial, a dysfunction of ETC could lead to increased symptoms, presumably due to increased ROS rather than decreased astrocytic ATP. It is likely that later in the pathology, the impact on microglia will be greater. Supporting this idea, an analysis of protein expression in the cortex of another mouse model showed that 42% and 25% of the proteins involved were specific to microglia and astrocytes, respectively^17^. Thus, dysregulation of the rhythmic functions of astrocytes could therefore be involved in neuronal disruption, and consequently affect hippocampal function, as has been shown in the SCN^49^.

Several scientific and technical limitations should be acknowledged. It is important to note that our study was only carried out at two time points (ZT2 and ZT14). A loss of rhythmic activity cannot be fully concluded, as phase shifts may explain our observations. However, whether involving reduced rhythmic variations or mere phase shifts, the consequences remain significant, since synchronization between the hippocampus and the surrounding world (light-dark rhythm, for example) via the SCN is altered. Furthermore, it is likely that the 8% of proteins showing self-tuning underestimates the number of proteins involved in daily variations, and only a finer analysis over time will confirm this value. However, in the ventral hippocampus, with measurements taken every 4 h, it has been shown that 10% of proteins show a time-dependent fluctuation^2^. In this study, only female mice were used, as they exhibit more pronounced pathology at the same age compared to males^59–63^. This greater vulnerability in females is also observed in humans, where women are significantly more predisposed than men to developing AD^64–68^. Since the estrous cycle was not considered, differences in cycle stages may have impacted our results. Our findings should also be confirmed in males. Our data were obtained from protein samples digested with trypsin, one of the most used approaches^69^. Although we cannot exclude the possibility of incomplete extraction, the comparable recovery rates between 3xTgAD and WT animals suggest that any bias was likely consistent across groups. Finally, our interpretations are based on the abondance of a relatively limited number of proteins (and not on their activity) and should therefore be considered with caution.

In conclusion, our data reveal a dysregulation in protein expression dynamics between ZT2 and ZT14 in 3xTgAD mice compared to WT. The transgenic animals show only about half of the time-dependent DEP from WT, and even less for astrocytes, indicating a reduced rhythmicity. All these observations were made at an early stage in the pathology, marked by the presence of an inflammatory reaction, the presence of circulating forms of Aβ and low extracellular Aβ deposits in the hippocampus^15,70^. Thus, our data show that early hippocampal dysfunction is time-dependent and mainly impacts the regulation of mitochondrial energy metabolism, repercussing on neurons and astrocytes. This dysfunction is marked by a decrease at ZT2 in proteins of the electron transport chain, notably complex I, suggesting a dysregulation of ATP production which may contribute to deficits in hippocampal function in AD.

## Methods

### Animals and sample collection

Female wild-type (WT) and 3xTgAD mice on a C57BL/6 genetic background, aged 7 months were reared under standard light/dark conditions (LD12:12) with *ad libitum* access to food and water. Animals were euthanized under isoflurane anesthesia by exsanguination via intracardiac perfusion with saline. Brains were removed, at ZT2 and ZT14. The 24 mice were randomly assigned to ZT2 and ZT14 (*n* = 6/ZT and genotype). We isolated the hippocampus for protein extraction. The samples were frozen in liquid nitrogen and then stored at −80 °C. Experimental authorization was obtained from the cantonal veterinary affairs service (SCAV) of the canton of Geneva (Switzerland) after approbation of the cantonal commission for animal experiments (CCEA) of the canton of Geneva (Switzerland). Methods were performed in accordance with relevant guidelines and regulations and data are reported in accordance with Animal Research: Reporting In Vivo Experiments (ARRIVE) guidelines.

### Proteomic

Hippocampi were sonicated after immersion in a solution of triton (50mM Tris HCl, 150mM NaCl, 1%Triton x100, protease and phosphatase inhibitors 1x, pH = 7.4). Following a 20 000 g centrifugation (20 min, 4 °C), the supernatants were collected. The total amount of proteins was measured by protein assay using the BCA kit. Proteins were precipitated and then digested with trypsin. Peptides were analyzed by nanoLC-MSMS using a vanquish NEO liquid chromatography system (Thermo Fisher Scientific) coupled with an Orbitrap Fusion Lumos Mass Spectrometer (Thermo Fisher Scientific). Raw files were imported into MaxQuant v2.6.7.0 in Standard mode, each annotated with genotype and Zeitgeber time. Label-free quantification was performed with FastLFQ (peptide correlation = on; minimum ratio count = 2), and the “Require values for valid LFQ” option was left ON so that only proteins quantified in all 24 runs were reported. Peptides were searched using Trypsin/P with up to one missed cleavage, carbamidomethylation (C) as a fixed modification, and methionine oxidation (M) as a variable modification. Standard target-decoy searching controlled the false discovery rate (FDR) at 1% for both peptides and proteins. The number of detected peptides per individual did not differ between groups (Supp. Data 11).

### Data processing with RStudio

Pre-processed data from the MaxQuant software were imported into R 4.4.2 and filtered with dplyr 1.1.4 to remove contaminants, reverse hits and “only identified by site” entries. Intensity columns were extracted, negative values were set to NA, duplicate UniProt IDs were collapsed by mean, log₂(x + 1) transformed, and quantile-normalized using limma 3.62.2. Sample metadata (genotypes 3xTgAD and WT, times ZT14 and ZT2) were handled with stringr 1.5.1 and tibble 3.3.0. Differential expression for each contrast (WT_ ZT14 vs. WT_ ZT2, AD_ ZT14 vs. AD_ ZT2) was performed via lmFit + eBayes in limma, and proteins with a |log₂FC| > 0.58 (corresponding to an absolute fold change of 1.5) were classified as DEPs. Significance threshold was set at a p-value of 0.05. EnhancedVolcano 1.24.0 was used to generate volcano plots, pheatmap 1.0.13 to display row-scaled (z-score) DEP expression as a clustered heatmap, and VennDiagram 1.7.3 to illustrate DEP overlaps. Figures were plotted with ggplot2 3.5.2. Functional enrichment analysis was carried out using with the reactome pathway 2024 database based on genes identified by DEP. Genes selected as cell-type markers for the ZT2 vs. ZT14 difference in WT were validated against the Brain RNA-Seq database (brainrnaseq.org). Cell-type comparisons between 3xTg-AD and WT were performed using the PanglaoDB database on proteins identified as DEP.

### Annotation and clustering analysis via DAVID and STRING

Functional enrichment and annotation clustering analysis were performed using the database for annotation, visualization and integrated discovery (DAVID) online tool^71^. Using the cluster 1 identified by the DAVID annotation, protein–protein interactions (Markov Cluster Algorithm, MCL, clustering) was retrieved via the search tool for the retrieval of interacting genes/proteins (STRING) online database^72^. Interactions were filtered using an inflation parameter of 3, favoring smaller, more specific clusters, and a minimum interaction score of 0.4, corresponding to the default medium-confidence threshold for biologically relevant interactions.

## Supplementary Information

Below is the link to the electronic supplementary material.

Supplementary Material 1

## Data Availability

The raw data can be accessed in the Yareta data repository using the following link: https://doi.org/10.26037/yareta: e7jnulvskbdr5ivjljzgt4qeyu.
